# Identification of metabolites from tandem mass spectra with a machine learning approach utilizing structural features

**DOI:** 10.1093/bioinformatics/btz736

**Published:** 2019-10-12

**Authors:** Yuanyue Li, Michael Kuhn, Anne-Claude Gavin, Peer Bork

**Affiliations:** 1 Structural and Computational Biology Unit, European Molecular Biology Laboratory, 69117 Heidelberg, Germany; 2 Molecular Medicine Partnership Unit (MMPU), 69117 Heidelberg, Germany; 3 Max Delbrück Center for Molecular Medicine, 13125 Berlin, Germany; 4 Department of Bioinformatics, Biocenter, University of Würzburg, 97074 Würzburg, Germany

## Abstract

**Motivation:**

Untargeted mass spectrometry (MS/MS) is a powerful method for detecting metabolites in biological samples. However, fast and accurate identification of the metabolites’ structures from MS/MS spectra is still a great challenge.

**Results:**

We present a new analysis method, called SubFragment-Matching (SF-Matching) that is based on the hypothesis that molecules with similar structural features will exhibit similar fragmentation patterns. We combine information on fragmentation patterns of molecules with shared substructures and then use random forest models to predict whether a given structure can yield a certain fragmentation pattern. These models can then be used to score candidate molecules for a given mass spectrum. For rapid identification, we pre-compute such scores for common biological molecular structure databases. Using benchmarking datasets, we find that our method has similar performance to CSI: FingerID and those very high accuracies can be achieved by combining our method with CSI: FingerID. Rarefaction analysis of the training dataset shows that the performance of our method will increase as more experimental data become available.

**Availability and implementation:**

SF-Matching is available from http://www.bork.embl.de/Docu/sf_matching.

**Supplementary information:**

Supplementary data are available at *Bioinformatics* online.

## 1 Introduction

Untargeted mass spectrometry (MS/MS) is a common approach for identification of metabolites in biological samples (Beger *et al.*, 2016; O’Kell *et al.*, 2017; Schrimpe-Rutledge *et al.*, 2016). Thereby, a complex biological sample is analyzed with liquid chromatography electrospray ionization tandem MS/MS, generating several thousands of MS/MS spectra in a few minutes. Inferring all molecular structures from these spectra in a fast, precise manner is, however, still a challenge. The currently fastest way of analyzing such data is to match fragmentation spectra of unknown substances to a reference spectral library (Kind *et al.*, 2018). These spectral libraries are usually built from known purified metabolites or generated by researches experiments. Some databases like METLIN (Guijas *et al.*, 2018), GNPS (Wang *et al.*, 2016) and Massbank (Horai *et al.*, 2010) are collecting these data. This experimental approach has the highest accuracy, however, generating these reference libraries is money- and time-consuming.

Several methods like MS2LDA (van der Hooft *et al.*, 2016, 2017), ChemFrag (Schüler *et al.*, 2018) and ChemDistiller (Laponogov *et al.*, 2018) have been developed for the prediction of functional chemical groups from metabolite spectra. Methods like XCMS2 (Benton *et al.*, 2008) use a similarity-based search to detect possible structural motifs of unknown metabolites in spectra. Also, many methods have been proposed for *in silico* metabolite identification from spectra (Heinonen *et al.*, 2012; Hummel *et al.*, 2010; Nguyen *et al.*, 2018; Vaniya and Fiehn, 2015). *In silico* fragmentation methods, such as ISIS (Kangas *et al.*, 2012), MS-FINDER (Tsugawa *et al.*, 2016), MetFrag (Ruttkies *et al.*, 2016) and CFM-ID (Allen *et al.*, 2015) strive to explain all fragment ions; these methods break every possible covalent bond, scoring each broken bond based on its strength. Some methods like CFM-ID can generate a pre-calculated spectral library. However, as molecular rearrangements occur during fragmentation, precise prediction of the rearrangement is very difficult, and these methods suffer from a low identification accuracy (Blaženović *et al.*, 2018). Other approaches like CSI: FingerID (Brouard *et al.*, 2016; Dührkop *et al.*, 2015, 2019; Ludwig *et al.*, 2018) convert a spectrum into a fragmentation tree, search this fragmentation tree against a database of known trees, and then infer a molecular fingerprint. These methods need to search fragmentation trees one by one in an online way, which makes it time-consuming when analyzing many spectra.

Depending on the type of biological sample, different chemical search spaces can be used. For well-studied sample types, such as cultured cells or human plasma, many of the metabolites in these samples have been analyzed before. In these cases, spectra can often be matched to reference libraries. Even if reference spectra are not available, there is a high chance that the compounds are part of curated databases such as the KEGG pathway database (Kanehisa *et al.*, 2017) or the HMDB database of metabolites (Wishart *et al.*, 2018). For such compounds, several identification methods have be developed (Blaženović *et al.*, 2018). More complex samples such as those from plants or the environment contain many molecules whose structures have not been determined yet, hampering compound identification using MS.

Here, we describe a new method called SF-Matching (SubFragment-Matching) to predict likely peaks in tandem mass spectra for small molecules using a machine learning approach. Circumventing the complexities of accurately modeling the fragmentation processes and probabilities of bond breakage, our new approach relies on detecting ‘fragile’ substructures in the molecule. These enable us to derive the respective fragmentation patterns to achieve high identification accuracy of compounds from mass spectra.

Unlike similarity-based approaches, which try to annotate spectra with a few features and fail if an unknown metabolite has chemical modifications, SF-Matching can be used for precise identification of a molecule when it contains similar substructures to known metabolites. SF-Matching also does not treat molecular bonds like the *in silico* methods: it does not predict the chemical reaction leading to the fragmentation, it only breaks bonds to find the subfragments, then uses the chemical fingerprint to capture similar molecular structures. Furthermore, the approach described here appears complementary to the existing method CSI: FingerID as a combination with it achieves a much higher accuracy than either method on its own with only a small sensitivity decrease.

## 2 Materials and methods

### 2.1 Converting spectra to subfragments

Given a molecular structure, we search for molecular substructures first. We remove all possible bonds $b_{s,t}$ connecting atoms $a_{s}$ and $a_{t}$, with the exception of carbon–carbon bonds in the aliphatic chain and bonds within ring systems. Removing a bond yields two sub-molecules with molecular formulas $F_{s,t}$ and $F_{t,s}$. For each heavy atom $a_{i}$ in a molecule, the Merck Molecular Force Field (Halgren, 1996) atom type $T_{a_{i}}$ was determined and the bond type $T_{b_{s,t}}$ was defined as $T_{b_{s,t}}=\left(T_{a_{s}},\;T_{a_{t}}\right)$.

The molecule’s corresponding spectrum can be treated as a list of n peaks $\left\{P_{1},\;P_{2},\;\ldots ,\;P_{n}\right\}$, here $P_{i}=\left(m/z\;M_{i},\;\text{intensity}\;I_{i}\right)$, and the formula of a fragment ion $F_{i}$ can be determined from $M_{i}$. A mass-to-charge ratio $M_{i}$ may correspond to several molecular formulas $F_{i}$. In this situation, only the formulas that are a subset of the precursor ion are considered. If more than one formula meets this criterion, we include all of them with the same weight.

Then, we test for all $F_{s,t}$ if they are a subset of $F_{i}$. In this case, we can calculate the difference of the molecular formulas $\Delta_{i,s,t}=F_{i}-F_{s,t}$, and define the subfragment $S_{j}=\left(T_{b_{s,t}},\Delta_{i,s,t}\right)$. Therefore, each peak $P_{i}$ with intensity $I_{i}$ corresponds to a set of subfragments $\left\{\left(S_{1},I_{i}\right),\;\left(S_{2},I_{i}\right),\;\ldots ,\;\left(S_{n},I_{i}\right)\right\}$.

### 2.2 Generating models for the subfragments

To build the prediction models, we collected 16 110 molecules for positive ion, 5884 molecules for negative ion from the GNPS, MassBank and in-house databases (Horai *et al.*, 2010; Wang *et al.*, 2016) (Supplementary Tables S1 and S2). For each spectrum in the database, the intensity of the spectrum was normalized so that the sum over all peak intensities equals one. If one molecule has multiple spectra, they will be treated individually. The spectral peaks were converted to fragment substructures as described earlier. Models were then built for all fragment substructures that occurred in at least five molecules.

Machine learning methods have difficulties within imbalanced training sets. In our case, most molecules do not generate a certain subfragment. To address this imbalance, the training data for a random forest can be augmented with weights for the individual items in the training data. Spectra that contained a peak corresponding to the fragment substructure were assigned the weight equal to the peak’s normalized intensity ($I_{i})$. The sum of weights is therefore $w=\sum_{i}I_{i}$. For spectra that did not contain a peak corresponding to the fragment substructure were assigned equal weights such that their sum is w. That is, if there are n spectra without the peak, their weight is w/n.

For each molecule, stereoisomer information was removed, and an 8191-bit chemical fingerprint generated using RDKit Fingerprint (Greg Landrum). Then, an extra-trees classifier was built using the scikit-learn (Pedregosa *et al.*, 2012) with 100 trees, using the chemical fingerprints of the complete structure as features and the presence of the fragment substructure as class label. A model will be built if a subfragment existed in at least five molecules. In total, models were built for 1 227 627 subfragments. Each of these models predicts the probability $P\left(S_{i,x|}|C\right)$ that a peak corresponding to the subfragment $S_{i,x}$ occurs given the chemical fingerprint C.

### 2.3 Predicting possible peaks for a given molecule

Given a molecule, its chemical fingerprint C was calculated as described earlier. Furthermore, all possible peaks $P_{i}\;$and their corresponding subfragment $S_{i,x}$ were determined from the molecular structure. The molecule’s chemical fingerprint was then used to predict a probability for the existence of a peak, using the pre-built models for the subfragment. When several subfragment had associated models for a given peak, the highest probability of these was assigned to that peak.

$$P\left(P_{i}||C\right)=\underset{x}{\max}P\left(S_{i,x}||C\right)$$

### 2.4 Spectrum scoring

Given a spectrum with peaks $P_{i}$ normalized peak intensities $I_{i}$, the score for a molecule with chemical fingerprint C is calculated by summing over the peak probabilities, using the intensities as weights:

$$\sum_{i}I_{i}P(P_{i}|C)$$

Peaks are determined by searching for molecular formulas within a certain mass accuracy.

### 2.5 Consensus scoring

For consensus scoring, candidate molecules were scored both with our method as well with CSI: FingerID. A prediction was only accepted if both methods have the same top prediction. When there was a tie for the top prediction, no consensus prediction was recorded.

### 2.6 Performance evaluation

For the CASMI 2016 dataset, the results of CFM-ID were obtained from the author’s submission, the results of CSI: FingerID were calculated by Sirius 4.0. For the CASMI 2017 dataset, the results of CSI: FingerID were obtained from the author’s submission, the results of CFM-ID were calculated by SE-CFM-trained model. For the EMBL metabolomics core facility (EMBL-MCF) and GNPS datasets, the results of CFM-ID were calculated by SE-CFM-trained model, the results of CSI: FingerID were calculated by Sirius 4.0. If one spectrum had several top predictions, the tie was broken by randomization.

### 2.7 Data availability and reproducibility

The SF-Matching software and a tutorial can be accessed from http://www.bork.embl.de/Docu/sf_matching. The source code is available at https://git.embl.de/grp-bork/sf-matching. An interactive example of using SF-Matching can be accessed at https://doi.org/10.24433/CO.6279326.v2. We further deposited the software and pre-calculated spectra predictions for relevant biological molecules [taken from KEGG (Kanehisa *et al.*, 2017), HMDB (Wishart *et al.*, 2018), ChEBI (Hastings *et al.*, 2016) and ChEMBL (Gaulton *et al.*, 2017)] in Zenodo: http://doi.org/10.5281/zenodo.3345099.

## 3 Results

**Fig. 1.**
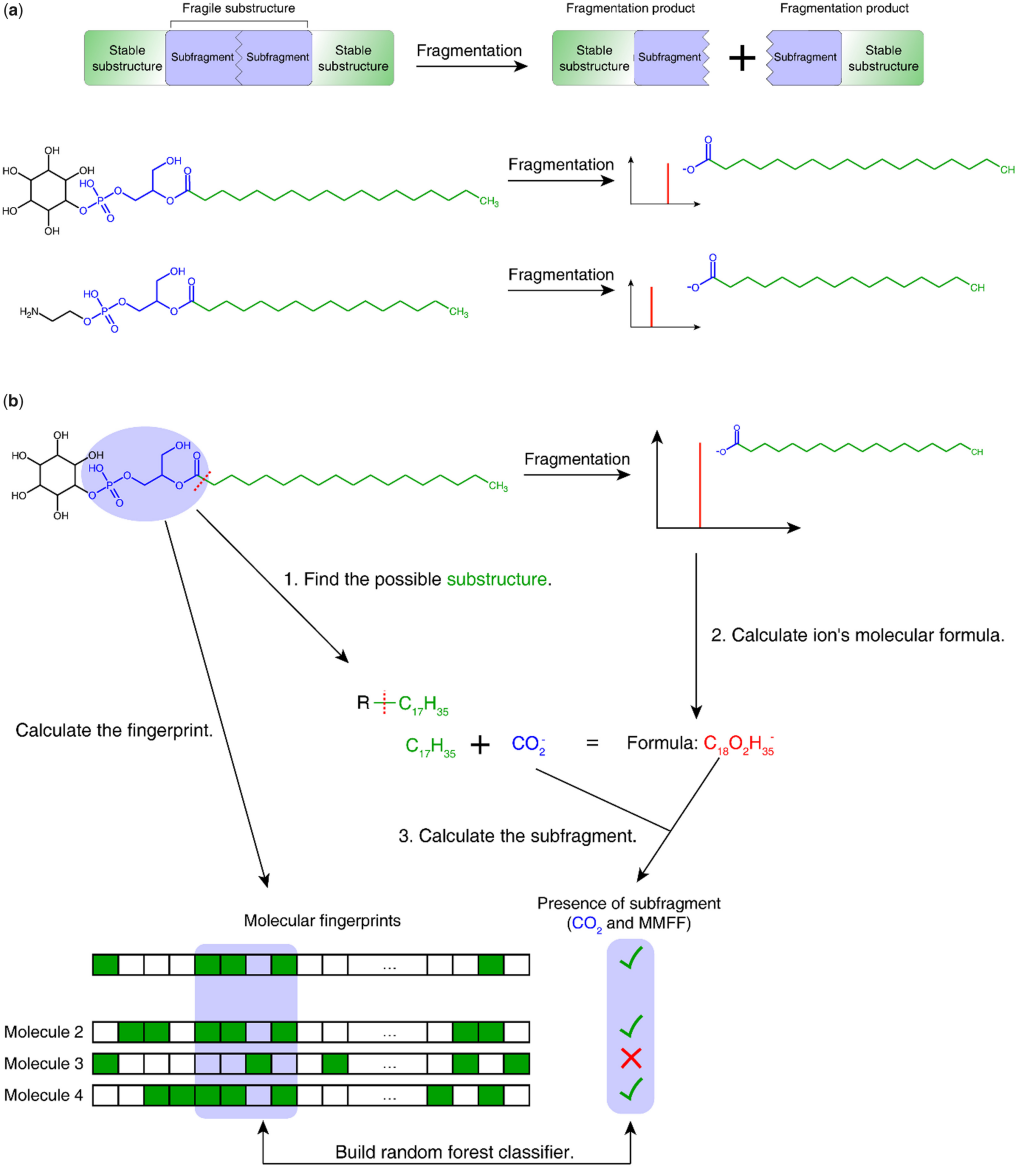
Schematic of the method. (**a**) The fragmentation of a molecule can be considered as breaking a fragile substructure surrounded by stable substructures. The resulting fragment contains both a stable substructure and part of the fragile substructure (which we term ‘subfragment’). (**b**) For training the method, we compare possible substructures to peaks in a reference database and calculate the subfragments that were observed. The presence of such a fragile subfragment can then be predicted based on the molecular fingerprint of the complete molecule

Our concept assumes that molecules consist of fragile and relatively stable substructures. Molecules with similar fragile structures will share similar fragmentation patterns even if they have different stable substructures. For example, in lysophosphatidylinositol and phosphatidylethanolamine, the inositol moiety, ethanolamine and the alkyl chain are stable substructures, connected by a fragile substructure containing ester bonds that are likely to lead to fragmentation. During fragmentation, the two different molecules with different alkyl chains will generate similar fatty acids as fragment ions, although the masses of their fragment ions are different (Fig. 1a). In the fatty acid that can be detected as a fragmentation product, the alkyl chain is the stable substructure and the carboxyl group is the fragment of the fragile substructure.

Our aim was therefore to develop a method that can detect the presence of fragile substructures and based on this predict if a given spectrum is likely to belong to a particular compound. We use machine learning to associate structural information contained in 2D chemical fingerprints with fragmentation patterns. Molecular fingerprints encode the presence of various substructures of the molecule in a vector of bits. If two molecules have similar fragile substructures, some bits of the fingerprint will be the same. As the substructure may vary from molecule to molecule, in our approach, we do not try to identify the extract fragile substructure, but instead use the fingerprint to represent it, and use machine learning to detect the predictive parts of the fingerprint. Given a training database of molecular structures and mass spectra, we process each spectrum individually. On a high level, training the model works as follows (Fig. 1b):

In the molecule associated with the spectrum, we find all substructures by individually breaking all covalent bonds in the molecules. For each broken bond, we record the resulting molecular formulas together with the bond type of the broken bond. For example, –C_17_H_35_ is one of the substructures of lysophosphatidylinositol.

For all peaks in the spectrum, we calculate the fragmentation products’ molecular formulas based on their m/z.

For each fragmentation product, we check which substructures found in Step 1 are a subset of the fragmentation product’s molecular formula. For any fragmentation product, there may be several such substructures. Considering each substructure individually, we then designate it as the stable substructure. The remaining part of the fragmentation product must therefore be a part of the fragile substructure, and its molecular formula can be determined from the formulas of the fragmentation product and the stable substructure. Together with the bond type information, we call this part of the fragile substructure a ‘subfragment’. Each molecule in the database can be checked if it contains a subfragment.

For each known molecule, we calculate its molecular fingerprint based on the structure of the complete molecule. After determining the presence of subfragments across all training spectra, random forest classifiers are trained for each subfragment based on the molecular fingerprint and the presence of the subfragment in each spectrum.

For testing if a certain molecule is likely to belong to a given spectrum, we do the reverse: we calculate its molecular fingerprint and find all possible subfragments based on the peaks of the spectrum. Then for every subfragment, we use the corresponding random forest classifier to predict the probability of the subfragment. After finding the possible molecular substructures, we can calculate the mass of all possible fragment ions by adding the formula of subfragment to the formula of substructure.

To increase the speed of the method, we pre-calculated the predicted spectra of all biomolecules in four databases that collect most of the known, relevant biological molecules [KEGG (Kanehisa *et al.*, 2017), HMDB (Wishart *et al.*, 2018), ChEBI (Hastings *et al.*, 2016) and ChEMBL (Gaulton *et al.*, 2017)]. This allows identification of compounds at a rate of more than 10 spectra per second on a laptop with a solid-state drive. The pre-calculated database and searching scripts can be downloaded from http://www.bork.embl.de/Docu/sf_matching.

**Fig. 2.**
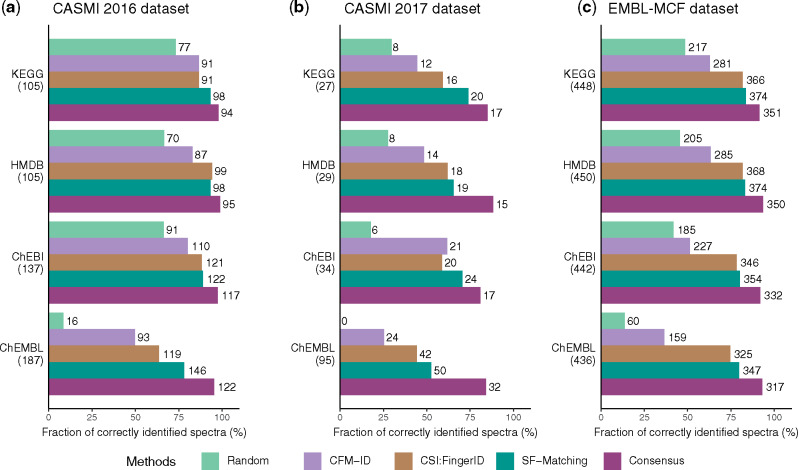
Performance evaluations when removing the test spectra from training dataset. The accuracy and the sensitivity of SF-Matching, and the consensus method are compared against random prediction and two established methods on (**a**) the CASMI 2016 dataset, (**b**) the CASMI 2017 dataset and (**c**) the EMBL-MCF dataset. The number in the parentheses indicates the total number of molecules which are contained in the various chemical databases; on right of the bar the number of correctly identified molecules is shown. Details on the ranking can be found in Supplementary Tables

We evaluated the performance of our approach by comparing it with CFM-ID (Allen *et al.*, 2015), and CSI: FingerID (Dührkop *et al.*, 2015), the two methods that showed very good performance in CASMI 2016 and can also run in batch mode (Schymanski *et al.*, 2017). As many of the test molecules also exist in those software’s training dataset, we remove the test spectra from our training dataset to achieve a fair comparison. First, we used all spectra provided in the context of the CASMI 2016 and 2017 automated structural identification challenge (Schymanski *et al.*, 2017) as benchmark dataset. To estimate the performance on multiple chemical databases, we limited the candidates to the molecules that are in the selected database. If one molecule was not present in the target database, the corresponding spectrum was not considered (Supplementary Table S3 and S4). In the CASMI 2016 dataset, SF-Matching had the best performance when searching against four different databases of known molecules (Fig. 2a). In the CASMI 2017 dataset, although the performances of all methods dropped, SF-Matching still showed better performance (Fig. 2b). As an additional benchmark, we also evaluated the methods using spectra from the EMBL-MCF spectral library (Palmer *et al.*, 2018; Supplementary Table S5). We selected candidate molecules from the respective chemical database with an m/z within 5 ppm of target molecules. In this dataset, SF-Matching had a better performance than CSI: FingerID and was also superior to CFM-ID (Fig. 2c).

**Fig. 3.**
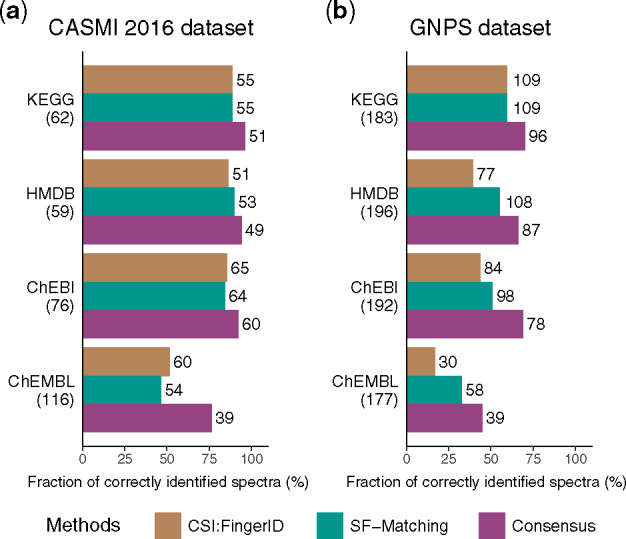
Performance evaluations when removing the test molecules from training dataset. The accuracy and the sensitivity of SF-Matching, and the consensus method is compared against CSI: FingerID on (**a**) the positive ion of CASMI 2016 dataset and (**b**) the GNPS dataset. The number in the parentheses indicates the total number of molecules which are contained in the various chemical databases; on right of the bar the number of correctly identified molecules is shown

For this and other approaches, the performance of *in silico* methods drops when removing not only test spectra, but all spectra for these molecules from the training dataset (Dührkop *et al.*, 2019). In CASMI 2016, the paper on CSI: FingerID reported its performance on positive ion when removing part of test molecules from the training dataset (Schymanski *et al.*, 2017). We removed all test molecules from our training dataset and rebuilt the model (Supplementary Table S6). In this dataset, our method has similar performance to CSI: FingerID (Fig. 3a). Based on the published list of CSI: FingerID’s training molecules, we also selected spectra from GNPS dataset whose corresponding spectra are not in CSI: FingerID’s training dataset (Supplementary Table S7). For this dataset, we also removed molecules from our training dataset and found that our method performed better than CSI: FingerID (Fig. 3b).

**Fig. 4.**
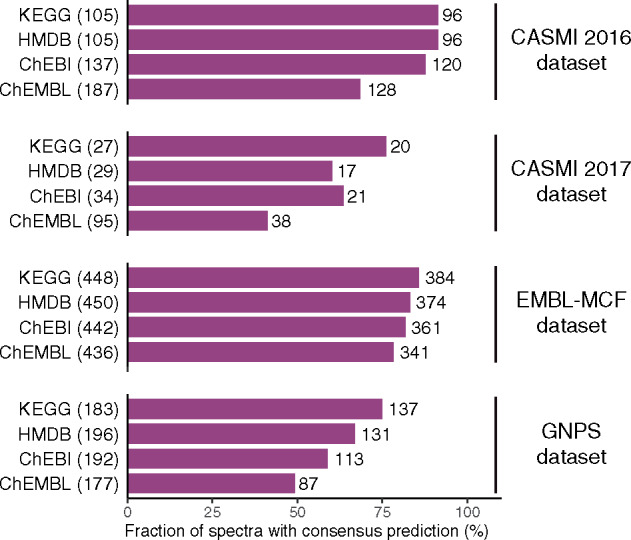
The fraction of spectra with consensus prediction. The number in the parentheses indicates the total number of molecules which are contained in the various chemical databases; on right of the bar the number of spectra with consensus prediction is shown

As our concept differs from existing ones, we reasoned that it should be possible to achieve a better accuracy if we combine prediction methods. As CSI: FingerID showed good performance in all the three benchmark dataset, we selected spectra where both our method and CSI: FingerID gave the same results. These consensus results achieved about 20% increase in accuracy than any single method, reaching >90% accuracy when analyzing the CASMI 2016 and EMBL-MCF datasets, and still >70% when analyzing the CASMI 2017 dataset. Due to the consensus calculation this comes at the cost of making predictions for fewer spectra, in average, around 55% spectra had consensus identification. The fraction varied between 40 and 80% in the CASMI 2016 and EMBL-MCF datasets and between 10 and 45% in CASMI 2017 (Fig. 4).

**Fig. 5.**
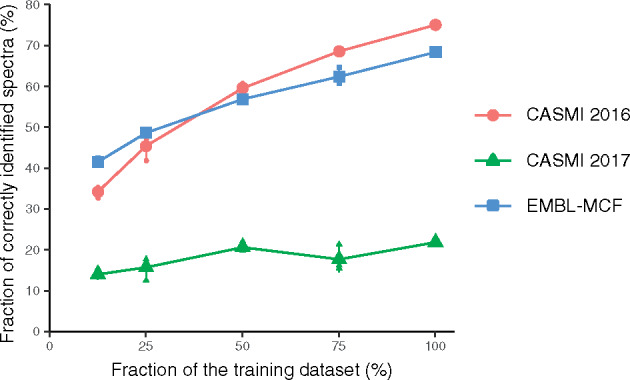
The effect of training dataset size on the performance on all datasets. For each training dataset size, three training datasets were randomly sampled from the origin whole dataset; here, we use the combination of four databases as candidate database

SF-matching uses structural features of molecules to build machine learning models and predict the probability that a given molecule spectrum gives rise to a measured spectrum. As machine learning approaches gain power with increasing training sets, we randomly selected subsets of the training dataset to evaluate the performance. Indeed, we observed an increase in the prediction performance with increased training set size (Fig. 5). This result suggests that SF-matching will increase performance with time with more experimental spectra becoming available. As shown in Supplementary Figure S1, the training data covers a wide range of the chemical space. Therefore, a general increase in the number of training molecules should be sufficient to increase the performance of SF-Matching.

Taken together, we have developed a new method called SF-Matching to identify the spectra of small molecules in biological samples. Depending on the goals of the MS experiments, SF-matching itself can be used to contribute to candidate molecule identification, given its stand- alone performance, but it can also be used in combination with CSI: FingerID for candidate predictions with high accuracy. Furthermore, as expected from machine learning techniques, the power of the method will increase in the future with the addition of diverse known spectra of biomolecules.

## Supplementary Material

btz736_Supplementary_Data

## References

[btz736-B1] Allen F. et al (2015) Competitive fragmentation modeling of ESI-MS/MS spectra for putative metabolite identification. Metabolomics, 11, 98–110.

[btz736-B2] Beger R.D. et al (2016) Metabolomics enables precision medicine: “A White Paper, Community Perspective”. Metabolomics, 12, 149. 27642271 10.1007/s11306-016-1094-6 PMC5009152

[btz736-B3] Benton H.P. et al (2008) XCMS2: processing tandem mass spectrometry data for metabolite identification and structural characterization. Anal. Chem., 80, 6382–6389. 18627180 10.1021/ac800795f PMC2728033

[btz736-B4] Blaženović I. et al (2018) Software tools and approaches for compound identification of LC-MS/MS data in metabolomics Metabolites, 8, 31. 10.3390/metabo8020031 PMC6027441 29748461

[btz736-B5] Brouard C. et al (2016) Fast metabolite identification with Input Output Kernel Regression. Bioinformatics, 32, i28–i36. 27307628 10.1093/bioinformatics/btw246 PMC4908330

[btz736-B6] Dührkop K. et al (2015) Searching molecular structure databases with tandem mass spectra using CSI: FingerID. Proc. Natl. Acad. Sci. USA, 112, 12580–12585. 26392543 10.1073/pnas.1509788112 PMC4611636

[btz736-B7] Dührkop K. et al (2019) SIRIUS 4: a rapid tool for turning tandem mass spectra into metabolite structure information. Nat. Methods, 16, 299–302. 30886413 10.1038/s41592-019-0344-8

[btz736-B8] Gaulton A. et al (2017) The ChEMBL database in 2017. Nucleic Acids Res., 45, D945–D954. 27899562 10.1093/nar/gkw1074 PMC5210557

[btz736-B10] Guijas C. et al (2018) Metabolomics activity screening for identifying metabolites that modulate phenotype. Nat. Biotechnol., 36, 316–320. 29621222 10.1038/nbt.4101 PMC5937131

[btz736-B11] Halgren T.A. (1996) Merck molecular force field. I. Basis, form, scope, parameterization, and performance of MMFF94. J. Comput. Chem., 17, 490–519.

[btz736-B12] Hastings J. et al (2016) ChEBI in 2016: improved services and an expanding collection of metabolites. Nucleic Acids Res., 44, D1214–D1219. 26467479 10.1093/nar/gkv1031 PMC4702775

[btz736-B13] Heinonen M. et al (2012) Metabolite identification and molecular fingerprint prediction through machine learning. Bioinformatics, 28, 2333–2341. 22815355 10.1093/bioinformatics/bts437

[btz736-B14] Horai H. et al (2010) MassBank: a public repository for sharing mass spectral data for life sciences. J. Mass Spectrom., 45, 703–714. 20623627 10.1002/jms.1777

[btz736-B15] Hummel J. et al (2010) Decision tree supported substructure prediction of metabolites from GC-MS profiles. Metabolomics, 6, 322–333. 20526350 10.1007/s11306-010-0198-7 PMC2874469

[btz736-B16] Kanehisa M. et al (2017) KEGG: new perspectives on genomes, pathways, diseases and drugs. Nucleic Acids Res., 45, D353–D361. 27899662 10.1093/nar/gkw1092 PMC5210567

[btz736-B17] Kangas L.J. et al (2012) In silico identification software (ISIS): a machine learning approach to tandem mass spectral identification of lipids. Bioinformatics, 28, 1705–1713. 22592377 10.1093/bioinformatics/bts194 PMC3381961

[btz736-B18] Kind T. et al (2018) Identification of small molecules using accurate mass MS/MS search. Mass Spectrom. Rev., 37, 513–532. 28436590 10.1002/mas.21535 PMC8106966

[btz736-B19] Laponogov I. et al (2018) ChemDistiller: an engine for metabolite annotation in mass spectrometry. Bioinformatics, 34, 2096–2102. 29447341 10.1093/bioinformatics/bty080 PMC9881669

[btz736-B20] Ludwig M. et al (2018) Bayesian networks for mass spectrometric metabolite identification via molecular fingerprints. Bioinformatics, 34, i333–i340. 29949965 10.1093/bioinformatics/bty245 PMC6022630

[btz736-B21] Nguyen D.H. et al (2018) Recent advances and prospects of computational methods for metabolite identification: a review with emphasis on machine learning approaches. Brief. Bioinform., bby066. 10.1093/bib/bby066 PMC6954430 30099485

[btz736-B22] O’Kell A.L. et al (2017) Untargeted metabolomic analysis in naturally occurring canine diabetes mellitus identifies similarities to human Type 1. Diabetes. Sci. Rep., 7, 9467. 28842637 10.1038/s41598-017-09908-5 PMC5573354

[btz736-B23] Palmer A. et al (2018) Curatr: a web application for creating, curating and sharing a mass spectral library. Bioinformatics, 34, 1436–1438. 29253079 10.1093/bioinformatics/btx786

[btz736-B24] Pedregosa F. et al (2012) Scikit-learn: machine Learning in Python. J. Mach. Learn. Res., 12, 2825–2830.

[btz736-B9] RDKit: Open-source cheminformatics; http://www.rdkit.org.

[btz736-B25] Ruttkies C. et al (2016) MetFrag relaunched: incorporating strategies beyond in silico fragmentation. J. Cheminform., 8, 3. 26834843 10.1186/s13321-016-0115-9 PMC4732001

[btz736-B26] Schrimpe-Rutledge A.C. et al (2016) Untargeted metabolomics strategies—challenges and emerging directions. J. Am. Soc. Mass Spectrom., 27, 1897–1905. 27624161 10.1007/s13361-016-1469-y PMC5110944

[btz736-B27] Schüler J.A. et al (2018) ChemFrag: chemically meaningful annotation of fragment ion mass spectra. J. Mass Spectrom., 53, 1104–1115. 30103269 10.1002/jms.4278

[btz736-B28] Schymanski E.L. et al (2017) Critical Assessment of Small Molecule Identification 2016: automated methods. J. Cheminform., 9, 22. 29086042 10.1186/s13321-017-0207-1 PMC5368104

[btz736-B29] Tsugawa H. et al (2016) Hydrogen rearrangement rules: computational MS/MS fragmentation and structure elucidation using MS-FINDER software. Anal. Chem., 88, 7946–7958. 27419259 10.1021/acs.analchem.6b00770 PMC7063832

[btz736-B30] van der Hooft J.J.J. et al (2016) Topic modeling for untargeted substructure exploration in metabolomics. Proc. Natl. Acad. Sci. USA, 113, 13738–13743. 27856765 10.1073/pnas.1608041113 PMC5137707

[btz736-B31] van der Hooft J.J.J. et al (2017) Unsupervised discovery and comparison of structural families across multiple samples in untargeted metabolomics. Anal. Chem., 89, 7569–7577. 28621528 10.1021/acs.analchem.7b01391 PMC5524435

[btz736-B32] Vaniya A., Fiehn O. (2015) Using fragmentation trees and mass spectral trees for identifying unknown compounds in metabolomics. Trends Analyt. Chem., 69, 52–61. 10.1016/j.trac.2015.04.002 PMC4509603 26213431

[btz736-B33] Wang M. et al (2016) Sharing and community curation of mass spectrometry data with Global Natural Products Social Molecular Networking. Nat. Biotechnol., 34, 828–837. 27504778 10.1038/nbt.3597 PMC5321674

[btz736-B34] Wishart D.S. et al (2018) HMDB 4.0: the human metabolome database for 2018. Nucleic Acids Res., 46, D608–D617. 29140435 10.1093/nar/gkx1089 PMC5753273

