# Supplementary material for: Identification of metabolites from tandem mass spectra with a machine learning approach utilizing structural features
Source: Bioinformatics. 2019 Oct 12;36(4):1213–8. doi: 10.1093/bioinformatics/btz736 (PMC7703789; doi:10.1093/bioinformatics/btz736)
Supplement: btz736_Supplementary_Data [file btz736_supplementary_data.zip › Supplemental figure 1.pptx]

## Slide 1
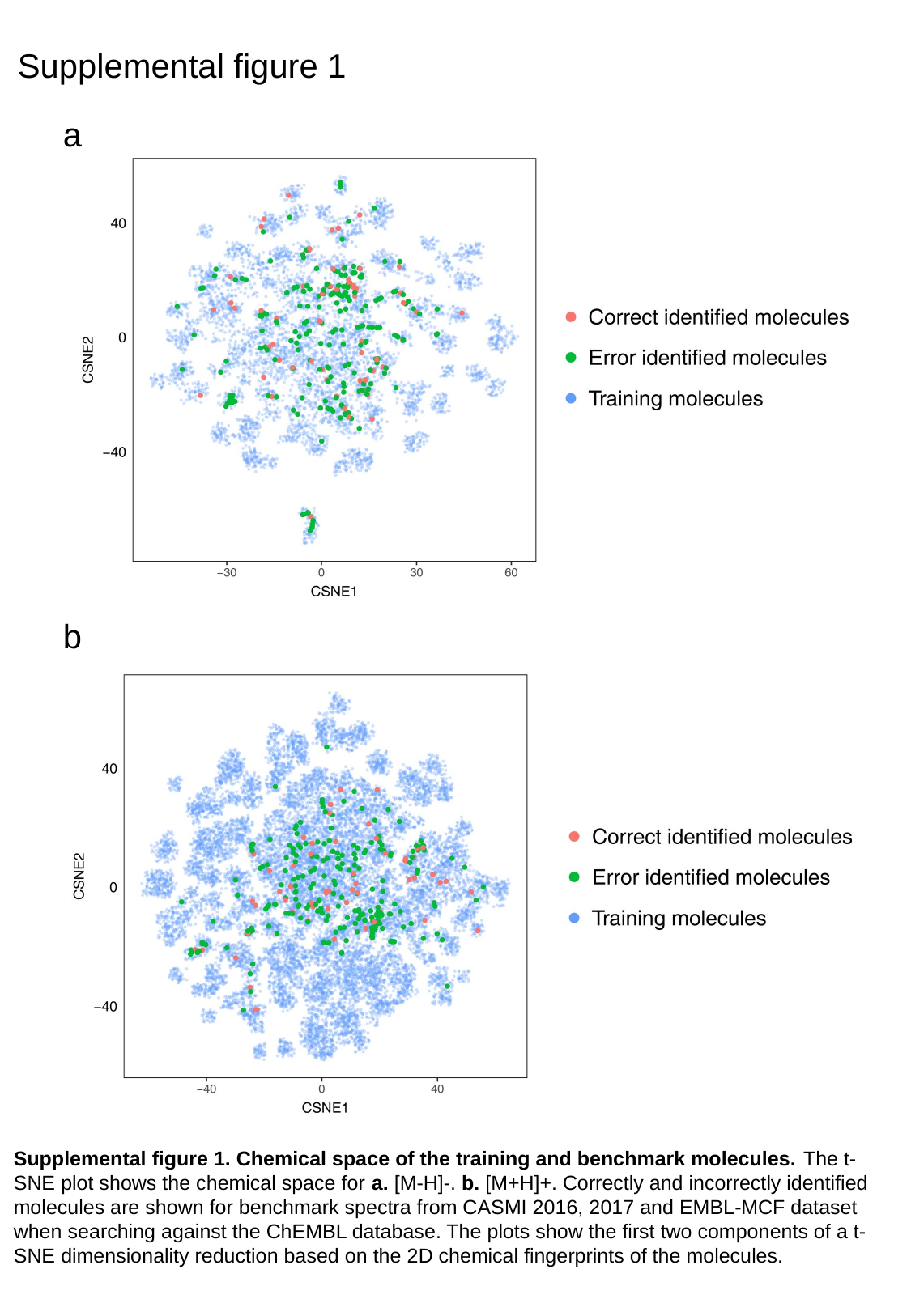

Supplemental figure 1
a
b
Supplemental figure 1. Chemical space of the training and benchmark molecules. The t-SNE plot shows the chemical space for a. [M-H]-. b. [M+H]+. Correctly and incorrectly identified molecules are shown for benchmark spectra from CASMI 2016, 2017 and EMBL-MCF dataset when searching against the ChEMBL database. The plots show the first two components of a t-SNE dimensionality reduction based on the 2D chemical fingerprints of the molecules.
